# Supplementary material for: Inhibition of lung tumorigenesis by transient reprogramming in cancer cells
Source: Cell Death Dis. 2024 Nov 25;15(11):857. doi: 10.1038/s41419-024-07207-2 (PMC11589828; doi:10.1038/s41419-024-07207-2)
Supplement: Supplementary file 1 — Supplemental Material [file 41419_2024_7207_MOESM1_ESM.pdf]

SUPPLEMENTAL INFORMATION

Inhibition of lung tumorigenesis by transient reprogramming in cancer cells

Pablo Pedrosa[1, †], Zhenguang Zhang[2, †], Victor Nuñez-Quintela[1, ††], David Macias[2, ††], Jianfeng Ge[2, ††], Mary Denholm[2, 3], Anna Dyas[2], Valentin Estevez-Souto[1], Patricia Lado-Fernandez[1, 4], Patricia Gonzalez[5], Maria Gomez[5], Jose Ezequiel Martin[6], Sabela Da Silva-Alvarez[1], Manuel Collado[1, 7, ‡, *] and Daniel Muñoz-Espín[2, 8, ‡, *]

** Corresponding authors:*
manuel.collado.rodriguez@sergas.es
dm742@cam.ac.uk

TABLE OF CONTENTS:

Fig. S1
Fig. S2
Fig. S3
Fig. S4
Fig. S5
Fig. S6
Fig. S7
Fig. S8
Fig. S9

Table S1

Running title: OSKM expression induces apoptosis and senescence in lung cancer

Classification: Biological Sciences

Keywords: reprogramming / lung cancer / apoptosis / senescence / pluripotency

Fig. S1

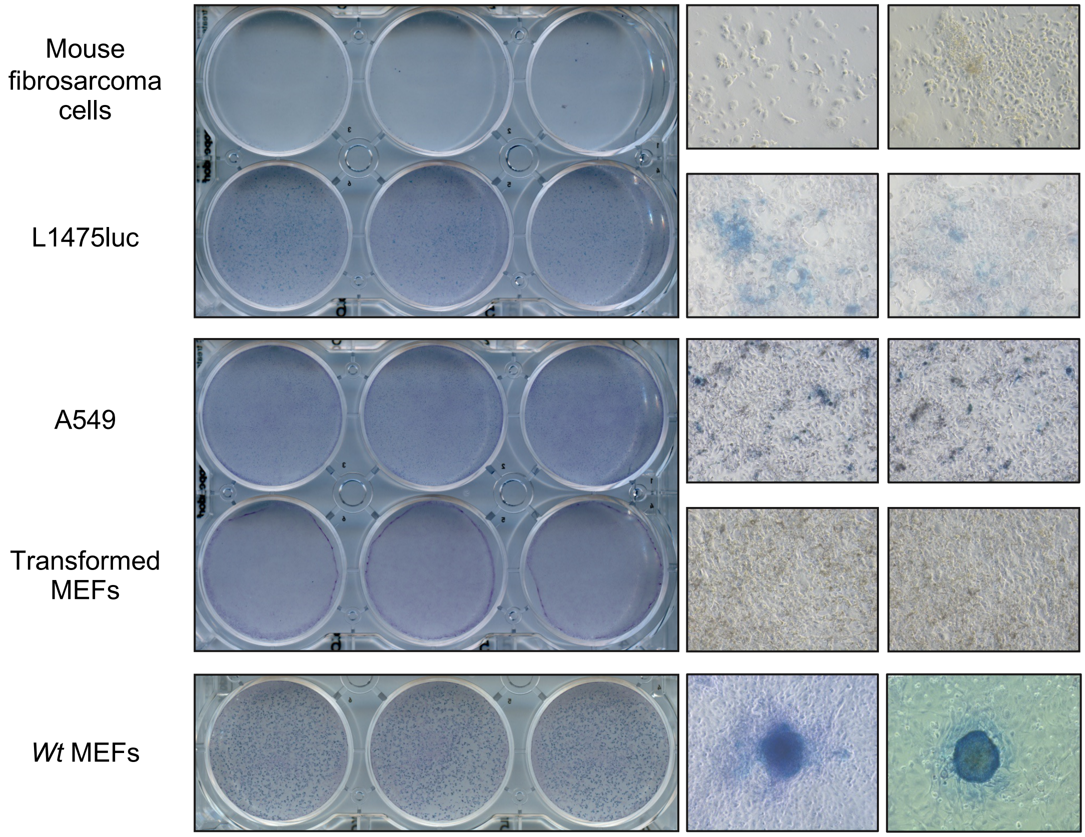
Mouse fibrosarcoma cells
L1475luc
A549
Transformed MEFs
Wt MEFs

Fig. S1. In vitro reprogramming of cancer cell lines and MEFs to iPSCs. (A) In vitro reprogramming assays of the indicated cellular types: mouse fibrosarcoma cells, mouse L1475luc and human A549 lung cancer cells, and transformed MEFs (carrying a construct that blocks p53 and another that causes an overexpression of the Ras oncogene). Wild-type (Wt) MEFs were used as positive reprogramming control to iPSCs. Alkaline phosphatase staining plates and representative microscopy images of cells after reprogramming are shown.

Fig. S2

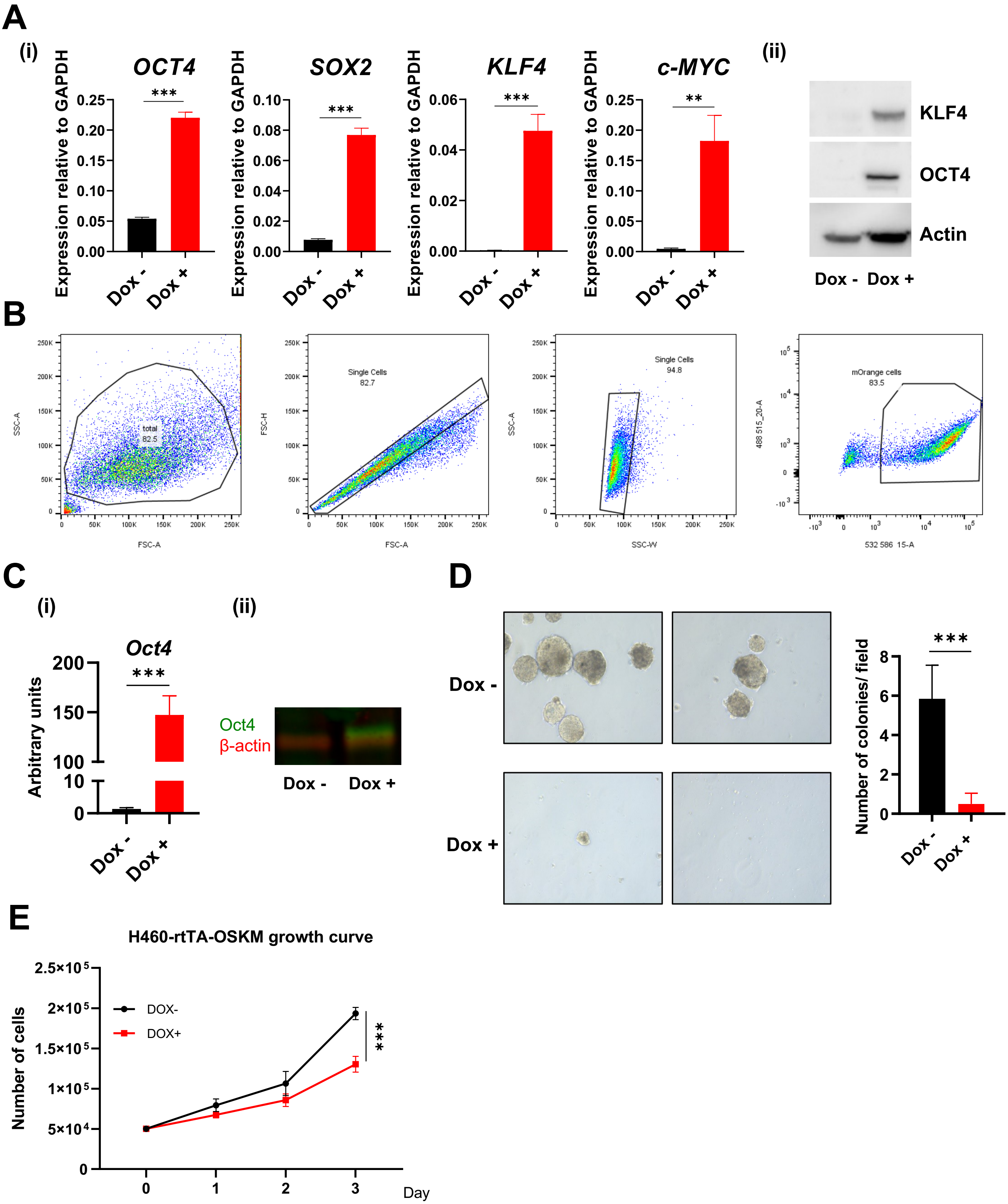
A
(i)
OCT4
SOX2
KLF4
c-MYC
Expression relative to GAPDH
***
**
Dox -
Dox +
(ii)
KLF4
OCT4
Actin
B
total
82.5
Single Cells
82.7
Single Cells
94.8
mOrange cells
83.5
SSC-A
FSC-A
FSC-H
SSC-W
488 515_20-A
532 586 15-A
C
(i)
(ii)
Oct4
Arbitrary units
Oct4
β-actin
D
Number of colonies/ field
E
H460-rtTA-OSKM growth curve
Number of cells
DOX-
DOX+
Day

Figure S2. Validation of the expression of reprogramming factors in A549 and L1475luc lung cancer cells. (A) mRNA levels of *Oct4*, *Sox2*, *Klf4* and *c-Myc* in A549-rtTA-OSKM cells, treated or not with doxycycline, by RT-qPCR (i), and KLF4 and OCT4 by Western blot (ii). **(B)** Flow cytometry plots showing the gating strategy for the sorting of L1475luc-rtTA-OSKM cells. The gating shows total cells, single cells, alive cells and mOrange positive cells in independent plots. **(C)** Expression levels of Oct4 in L1475luc-rtTA-OSKM cells, treated or not with 1 μg/ml doxycycline, by RT-qPCR (i), and Oct4 by Western blot (ii). Oct4 protein is shown in red color and β-actin in green color. **(D)** Representative image (left) and quantification (right) of a colony formation assay in soft agar of A549-rtTA-OSKM cells treated or not with doxycycline (1μg/ml). **(E)** Cell growth curves of H460-rtTA-OSKM cells, over 3 days, treated or not with doxycycline (1 μg/ml).

Statistical significance was calculated using Student's t-test, ***P<0.001; **P<0.01; *P<0.05. Data are mean ± SD.

Fig. S3

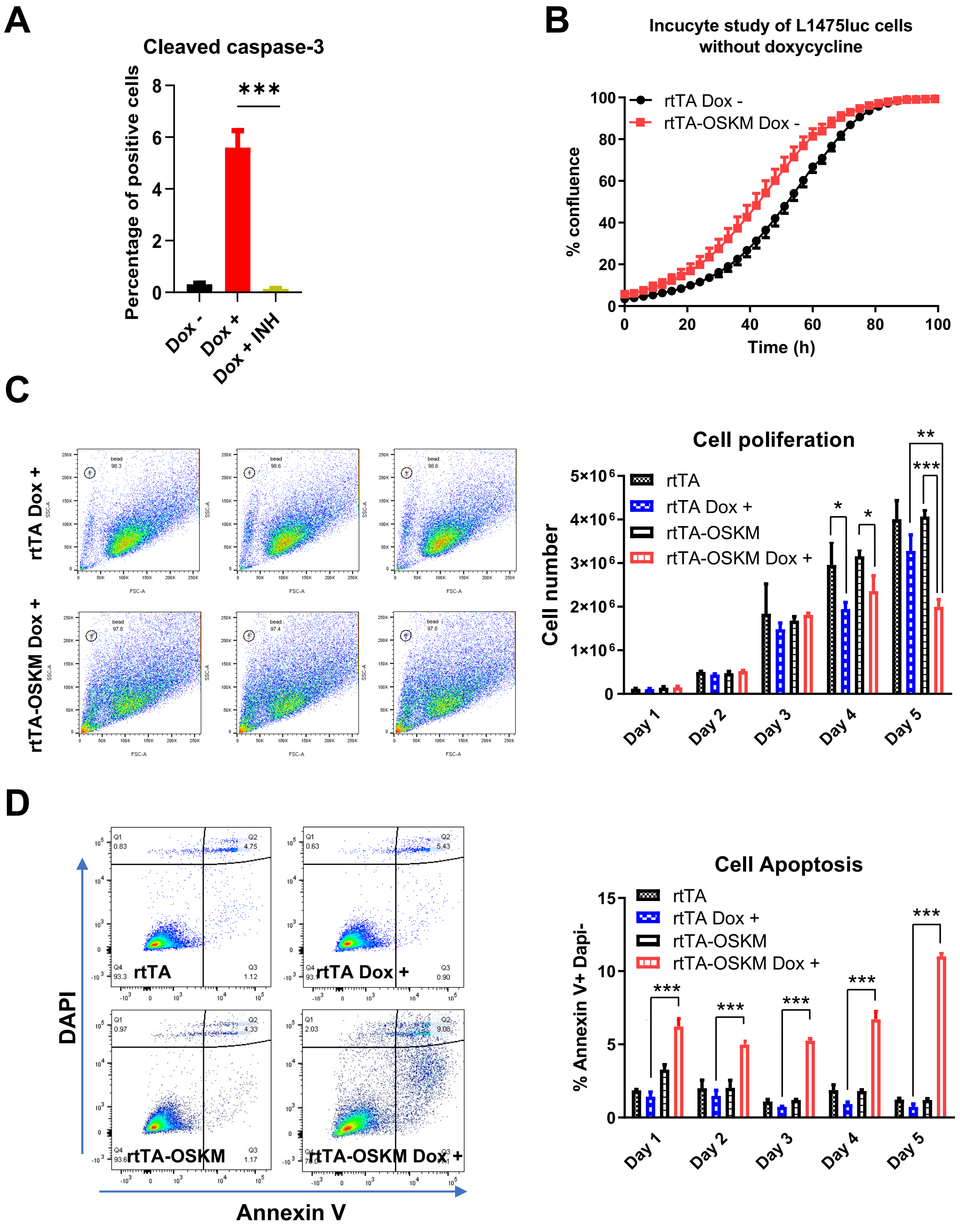
A
Cleaved caspase-3
Percentage of positive cells
***
Dox -
Dox +
Dox + INH
B
Incucyte study of L1475luc cells without doxycycline
rtTA Dox -
rtTA-OSKM Dox -
% confluence
Time (h)
C
rtTA Dox +
rtTA-OSKM Dox +
Cell poliferation
Cell number
rtTA
rtTA Dox +
rtTA-OSKM
rtTA-OSKM Dox +
Day 1
Day 2
Day 3
Day 4
Day 5
D
DAPI
Annexin V
rtTA
rtTA Dox +
rtTA-OSKM
rtTA-OSKM Dox +
Cell Apoptosis
% Annexin V+ Dapi-
rtTA
rtTA Dox +
rtTA-OSKM
rtTA-OSKM Dox +
Day 1
Day 2
Day 3
Day 4
Day 5

Fig. S3. OSKM expression induces lung cancer cell apoptosis and senescence in vitro. **(A)** Percentage of A549-rtTA-OSKM cells positive for cleaved caspase-3 expressing (Dox +) or not (Dox -) the reprogramming factors, and treated or not with the caspase inhibitor (INH), as indicated. **(B)** Quantification of L1475luc-rtTA and L1475luc-rtTA-OSKM cell proliferation in the absence of doxycycline using Incucyte technology. Images were taken every 3 h for over 96 h. **(C)** and **(D)** Flow cytometry analysis of L1475luc-rtTA and L1475luc-rtTA-OSKM cell proliferation and apoptosis in the absence or presence of doxycycline, as indicated. Left, flow cytometry plots. Right, quantification of cell numbers and percentage of Annexin V^{+}/$DAPI^{-}$ cells (Q3), as indicated.

Statistical significance was calculated using Student's t-test, ***$P<0.001$; **$P<0.01$; *$P<0.05$. Data are mean ± SD.

Fig. S4

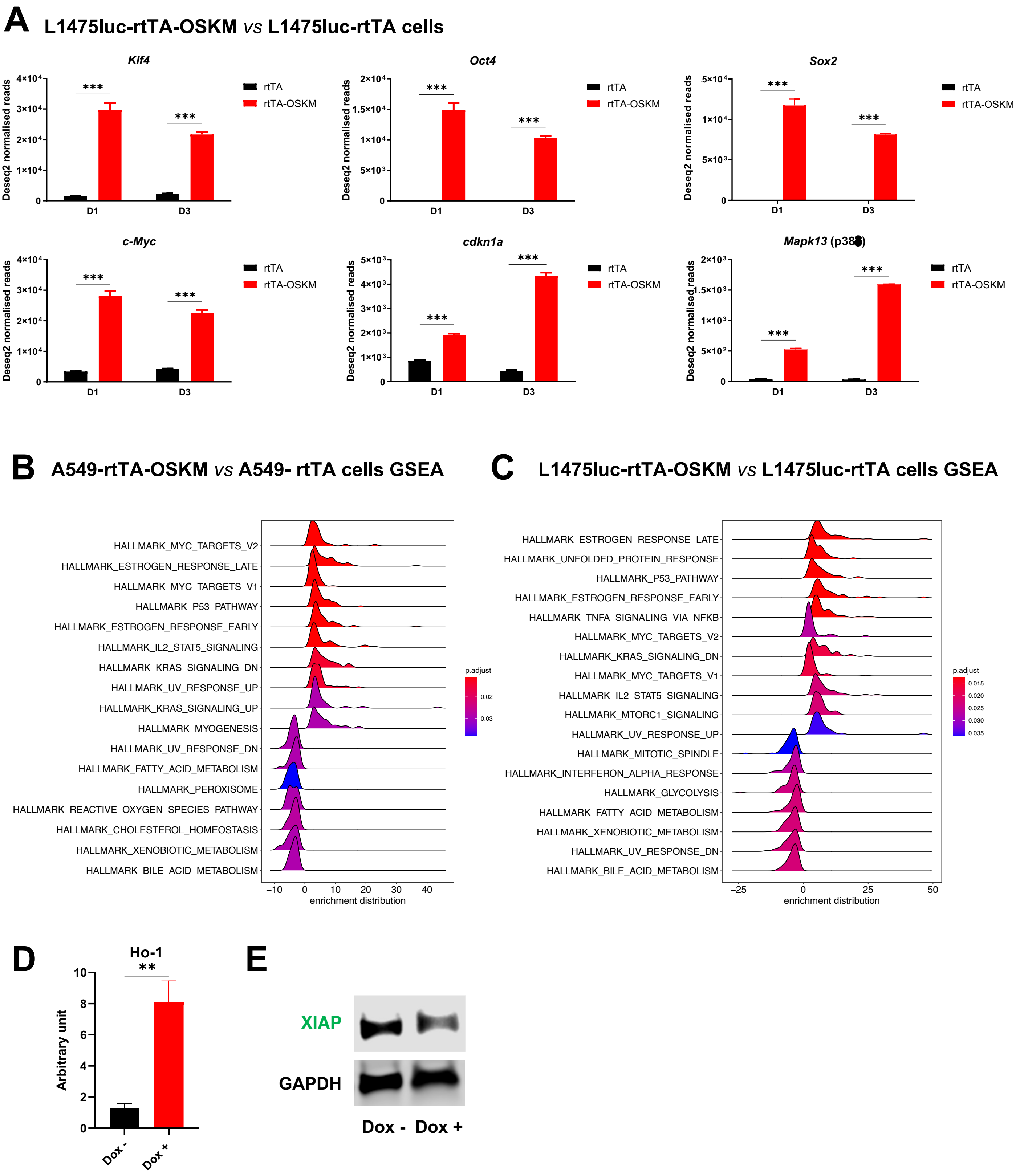

A
L1475luc-rtTA-OSKM vs L1475luc-rtTA cells
Klf4
Oct4
Sox2
c-Myc
cdkn1a
Mapk13
Deseq2 normalised reads
rtTA
rtTA-OSKM
D1
D3
***
B
A549-rtTA-OSKM vs A549- rtTA cells GSEA
HALLMARK_MYC_TARGETS_V2
HALLMARK_ESTROGEN_RESPONSE_LATE
HALLMARK_MYC_TARGETS_V1
HALLMARK_P53_PATHWAY
HALLMARK_ESTROGEN_RESPONSE_EARLY
HALLMARK_IL2_STAT5_SIGNALING
HALLMARK_KRAS_SIGNALING_DN
HALLMARK_UV_RESPONSE_UP
HALLMARK_KRAS_SIGNALING_UP
HALLMARK_MYOGENESIS
HALLMARK_UV_RESPONSE_DN
HALLMARK_FATTY_ACID_METABOLISM
HALLMARK_PEROXISOME
HALLMARK_REACTIVE_OXYGEN_SPECIES_PATHWAY
HALLMARK_CHOLESTEROL_HOMEOSTASIS
HALLMARK_XENOBIOTIC_METABOLISM
HALLMARK_BILE_ACID_METABOLISM
p.adjust
enrichment distribution
C
L1475luc-rtTA-OSKM vs L1475luc-rtTA cells GSEA
HALLMARK_ESTROGEN_RESPONSE_LATE
HALLMARK_UNFOLDED_PROTEIN_RESPONSE
HALLMARK_P53_PATHWAY
HALLMARK_ESTROGEN_RESPONSE_EARLY
HALLMARK_TNFA_SIGNALING_VIA_NFKB
HALLMARK_MYC_TARGETS_V2
HALLMARK_KRAS_SIGNALING_DN
HALLMARK_MYC_TARGETS_V1
HALLMARK_IL2_STAT5_SIGNALING
HALLMARK_MTORC1_SIGNALING
HALLMARK_UV_RESPONSE_UP
HALLMARK_MITOTIC_SPINDLE
HALLMARK_INTERFERON_ALPHA_RESPONSE
HALLMARK_GLYCOLYSIS
HALLMARK_FATTY_ACID_METABOLISM
HALLMARK_XENOBIOTIC_METABOLISM
HALLMARK_UV_RESPONSE_DN
HALLMARK_BILE_ACID_METABOLISM
p.adjust
enrichment distribution
D
Ho-1
**
Arbitrary unit
Dox -
Dox +
E
XIAP
GAPDH
Dox - Dox +

Fig. S4. Transcriptomic and proteomic profiles of lung cancer cells upon OSKM expression. **(A)** mRNA levels of *Oct4*, *Sox2*, *Klf4*, *c-Myc*, *Cdkn2a* and *Mapk13* in L1475luc-rtTA and L1475luc-rtTA-OSKM cells at day 1 or 3 post-doxycycline treatment, by RT-qPCR. **(B)** and **(C)** Ridge plot displaying the gene set enrichment analysis (GSEA) for A549-rtTA-OSKM *vs* A549-rtTA cells and L1475luc-rtTA and L1475luc-rtTA-OSKM with adjusted *p-value* <0.05. **(D)** mRNA levels of *Ho-1* in L1475luc-rtTA-OSKM cells treated or not with doxycycline, by RT-qPCR. **(E)** Western blot of XIAP expression levels in L1475luc-rtTA-OSKM cells expressing (Dox +) or not (Dox -) the reprogramming factors.

Statistical significance was calculated using Student's t-test, ***P<0.001; **P<0.01; *P<0.05. Data are mean ± SD.

Fig. S5

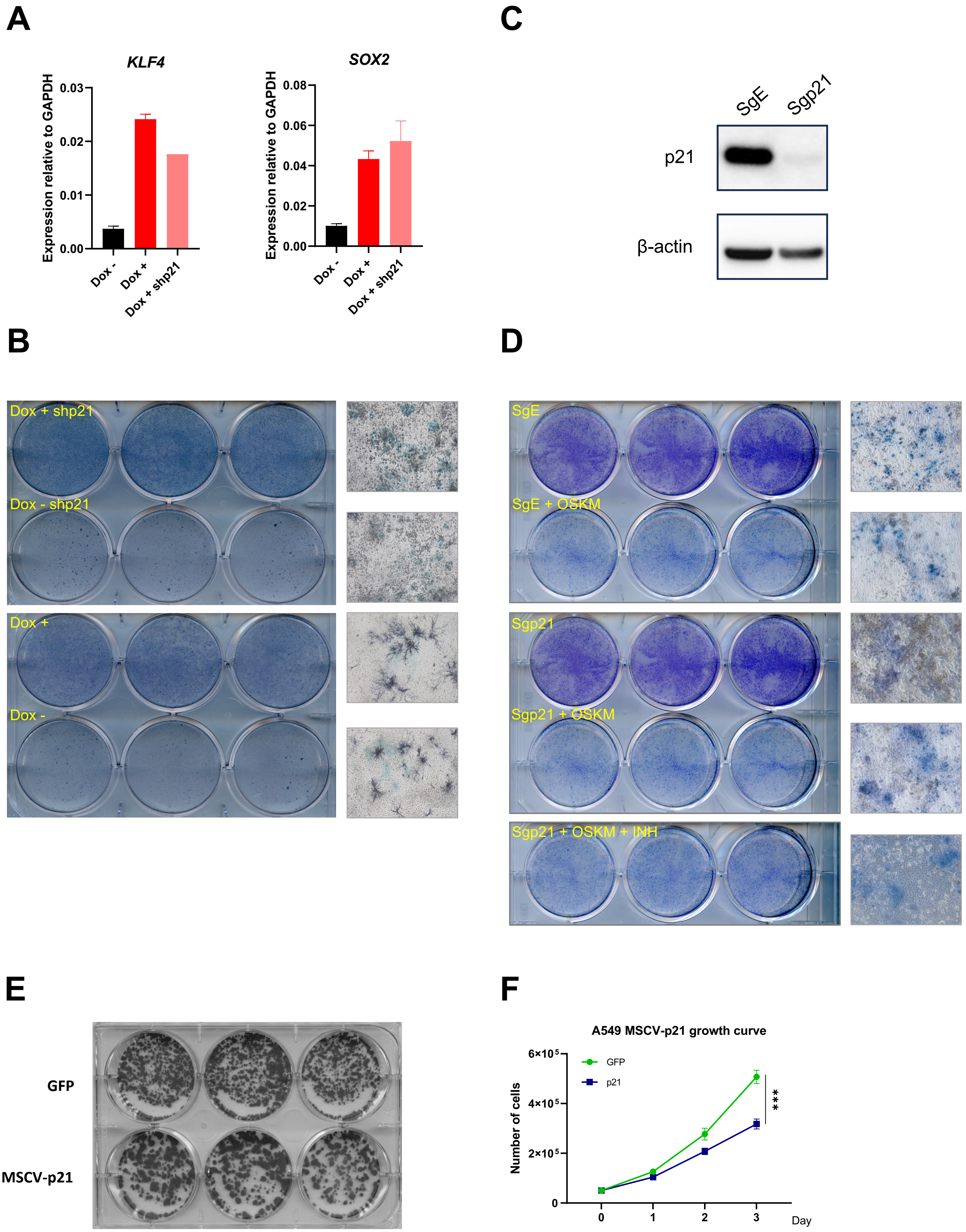

A
KLF4
Expression relative to GAPDH
0.03
0.02
0.01
0.00
Dox -
Dox +
Dox + shp21
SOX2
Expression relative to GAPDH
0.08
0.06
0.04
0.02
0.00
Dox -
Dox +
Dox + shp21
C
SgE
Sgp21
p21
β-actin
B
Dox + shp21
Dox - shp21
Dox +
Dox -
D
SgE
SgE + OSKM
Sgp21
Sgp21 + OSKM
Sgp21 + OSKM + INH
E
GFP
MSCV-p21
F
A549 MSCV-p21 growth curve
Number of cells
6×10^5
4×10^5
2×10^5
0
GFP
p21
***
0
1
2
3
Day

Fig. S5 (continued)

G

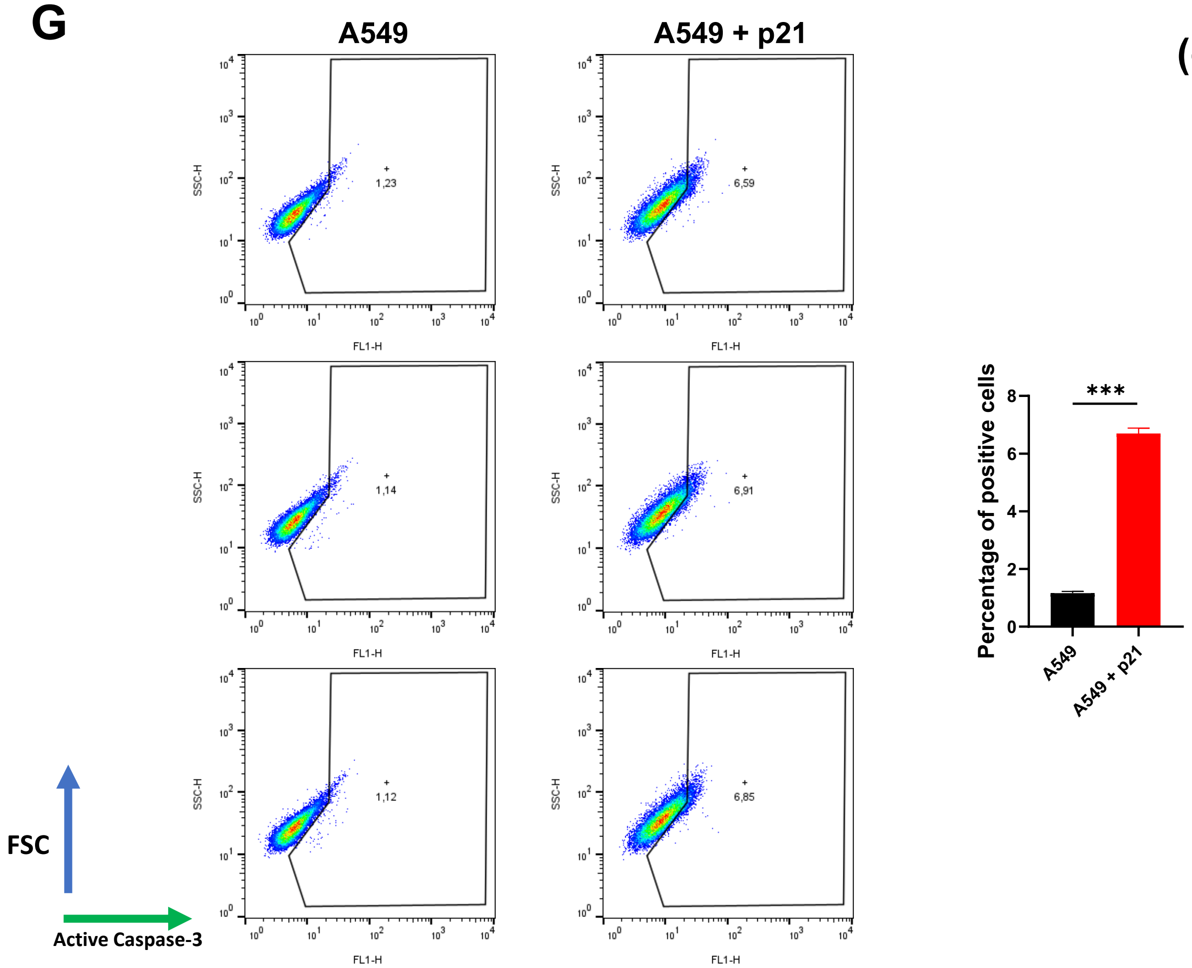

H

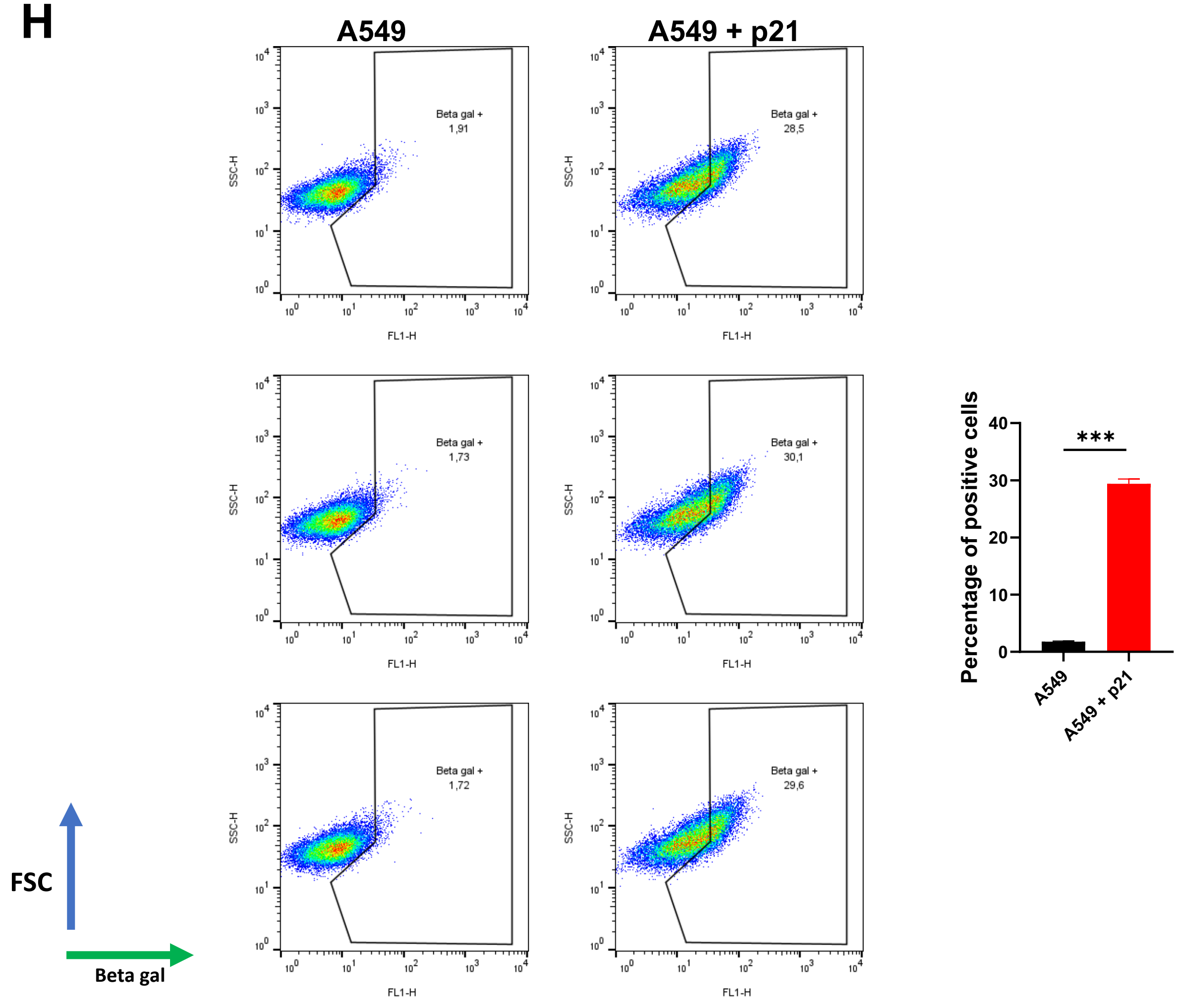

Fig. S5. p21 downregulation and knockdown do not allow A549 reprogramming to pluripotency. **(A)** mRNA expression levels of *KLF4* and *SOX2* by RT-qPCR in A549-rtTA-OSKM cells expressing (Dox +) or not (Dox -) the reprogramming factors, and in cells expressing the factors and after knockdown of *p21* (*CDKN1A*) (Dox + shp21). **(B)** In vitro reprogramming assays of A549 cells that do or do not express shp21. Alkaline phosphatase staining plaques and representative microscopy images of cells after reprogramming are shown. **(C)** Western blot for p21 protein levels after bleomycin treatment of A549 cells that express CRISPR/Cas9 with an empty guide (SgE) and with a guide against p21 (Sgp21). **(D)** In vitro reprogramming assays of A549 cells that express CRISPR/Cas9 with an empty guide (SgE), with a guide against p21 (Sgp21), or with a guide against p21 and treated with a pan caspase inhibitor (Sgp21 + INH). Alkaline phosphatase staining plaques and representative microscopy images of cells after reprogramming are shown. **(E)** Representative images of colony formation assays in A549 cells overexpressing or not p21. **(F)** Cell growth curves of in A549 cells overexpressing or not p21. **(G)** Quantification of active caspase-3 levels in A549 cells overexpressing or not p21. Plasmid: pMSCV-p21. **(H)** Quantification of SA-ß-gal activity levels in A549 cells overexpressing or not p21. Plasmid: pMSCV-p21

Statistical significance was calculated using Student's t-test, ***$P<0.001$; **$P<0.01$; *$P<0.05$. Data are mean ± SD.

Fig. S6

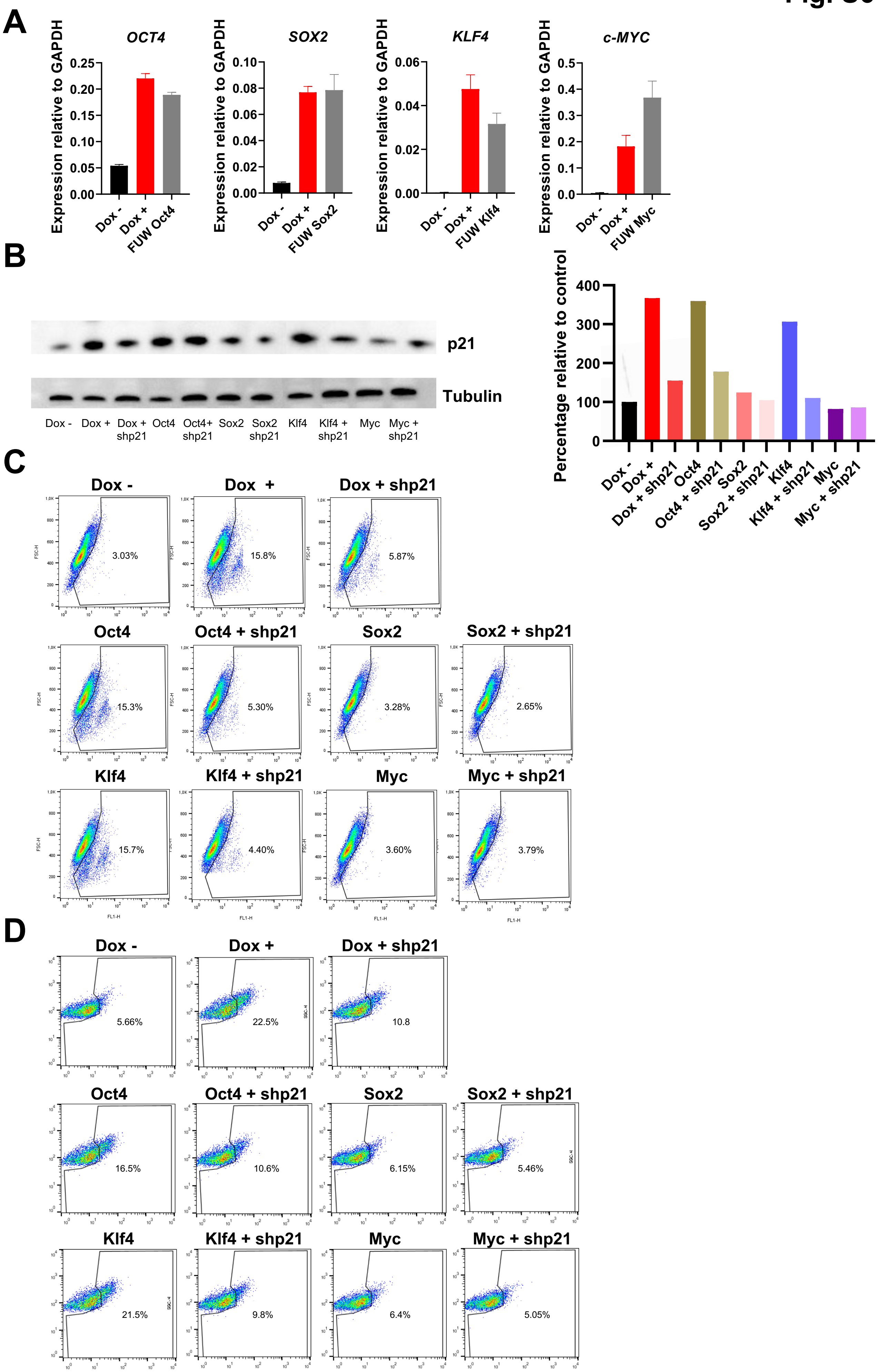
A
OCT4
SOX2
KLF4
c-MYC
Expression relative to GAPDH
Dox -
Dox +
FUW Oct4
FUW Sox2
FUW Klf4
FUW Myc
B
p21
Tubulin
Dox -
Dox +
Dox + shp21
Oct4
Oct4+ shp21
Sox2
Sox2 shp21
Klf4
Klf4 + shp21
Myc
Myc + shp21
Percentage relative to control
C
Dox -
3.03%
Dox +
15.8%
Dox + shp21
5.87%
Oct4
15.3%
Oct4 + shp21
5.30%
Sox2
3.28%
Sox2 + shp21
2.65%
Klf4
15.7%
Klf4 + shp21
4.40%
Myc
3.60%
Myc + shp21
3.79%
D
Dox -
5.66%
Dox +
22.5%
Dox + shp21
10.8
Oct4
16.5%
Oct4 + shp21
10.6%
Sox2
6.15%
Sox2 + shp21
5.46%
Klf4
21.5%
Klf4 + shp21
9.8%
Myc
6.4%
Myc + shp21
5.05%

Fig. S6. Contribution of individual reprogramming factors to the impaired cell growth and effect of p21 knockdown. **(A)** Expression of reprogramming factors by RT-qPCR in A549 cells that do not express reprogramming factors (Dox -), cells that co-express them (Dox +) and cells that individually express reprogramming factors (FUW OCT4, SOX2, KLF4 or c-MYC). **(B)** Expression of p21 protein levels in A549 cells that express or not the reprogramming factors (Dox +/-), in combination or individually (OCT4, SOX2, KLF4 and c-MYC), alone or after shp21 (+ shp21). Blot (left), quantification (right). **(C)** Flow cytometry plots for active caspase-3 as a marker indicative of cell apoptosis. **(D)** Flow cytometry plots for SA-β-gal as a marker indicative of cell senescence.

Fig. S7

A

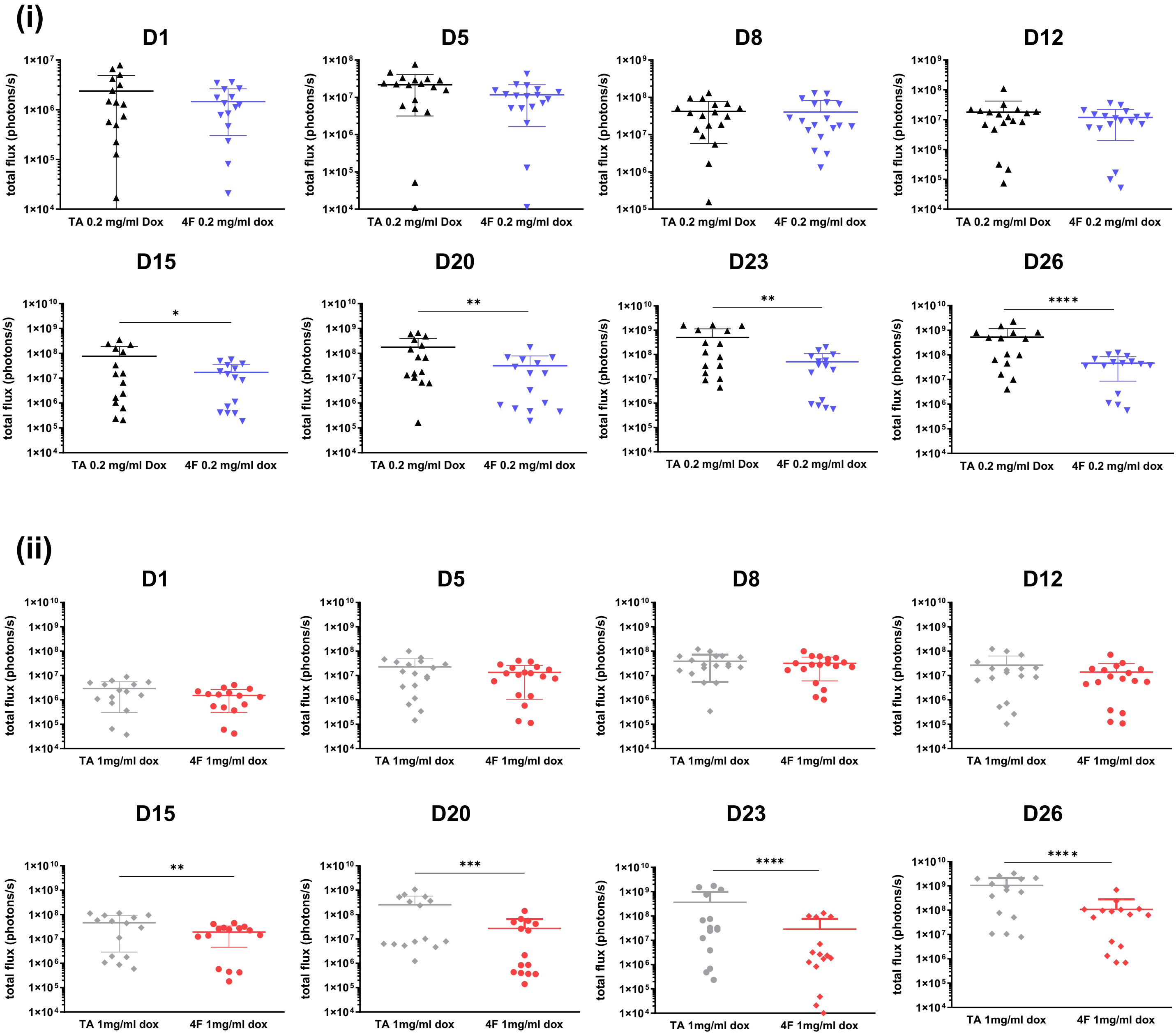
(i)
D1
D5
D8
D12
D15
D20
D23
D26
total flux (photons/s)
TA 0.2 mg/ml Dox
4F 0.2 mg/ml dox
*
**
**
****
(ii)
D1
D5
D8
D12
D15
D20
D23
D26
total flux (photons/s)
TA 1mg/ml dox
4F 1mg/ml dox
**
***
****
****

B

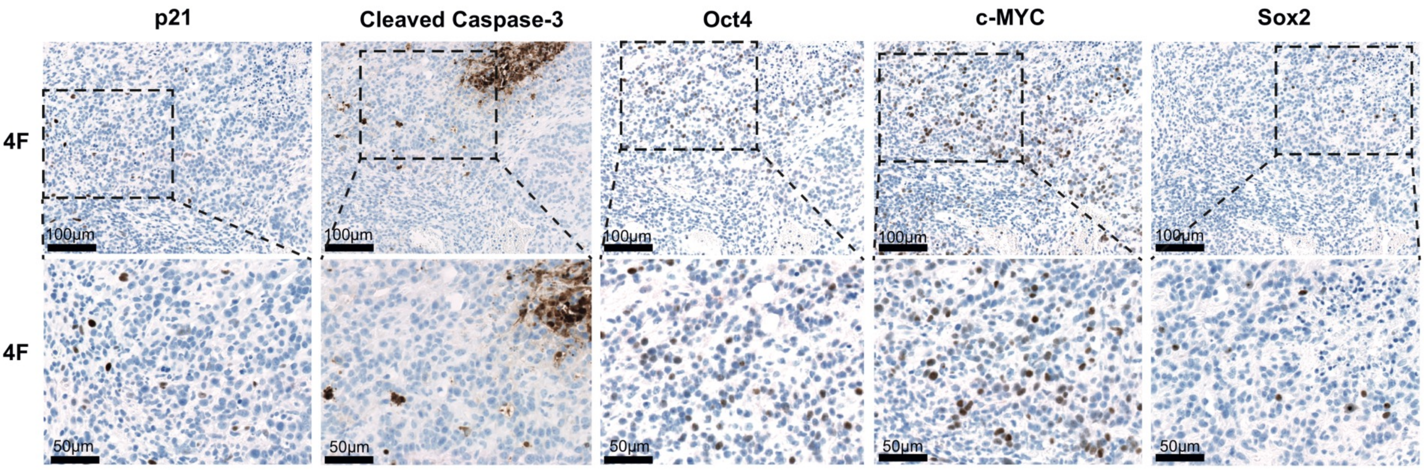
p21
Cleaved Caspase-3
Oct4
c-MYC
Sox2
4F
4F
100μm
50μm

Fig. S7. Expression of OSKM impairs growth of subcutaneous tumors. **(A)** Quantification of bioluminescence signal from groups treated with 0.2 mg/ml (i) and 1 mg/ml (ii) doxycycline. **(B)** Immunohistochemistry for p21, cleaved Caspase-3, Oct4, c-Myc and Sox2 in 4F mice.

Data shown are mean and SD for each individual day. Wilcoxon matched-pairs signed rank test is used to compare the 2 groups on each day. *, $p<0.05$; **, $p<0.01$; ****, $p<0.0001$

Fig. S8

A

(i) DOXYCYCLINE

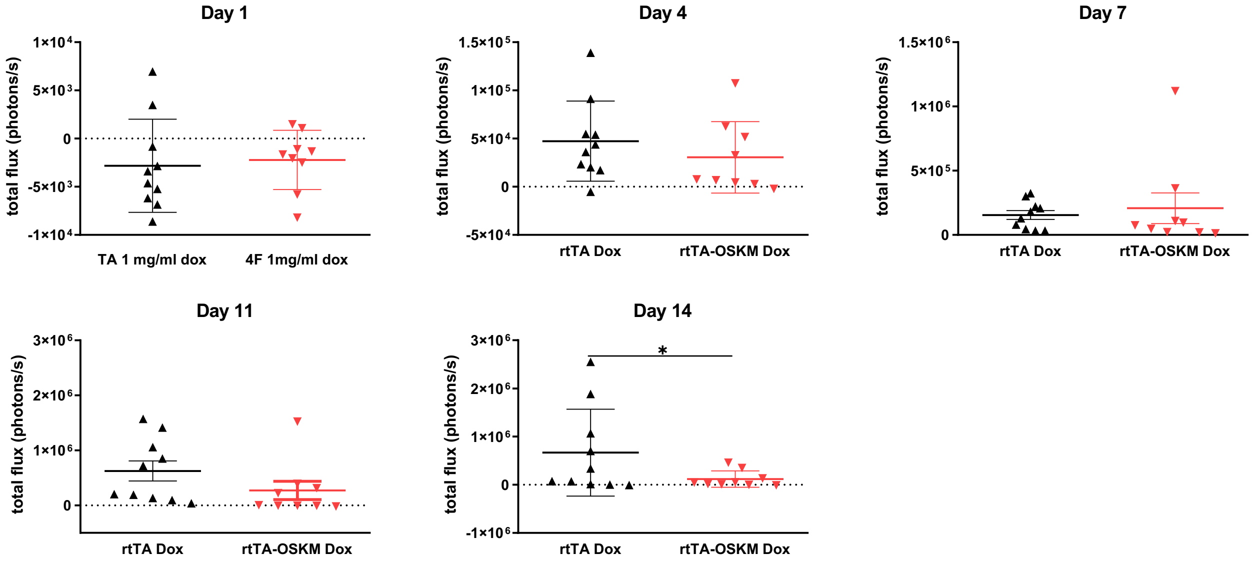

(i) CONTROL NO DOXYCYCLINE

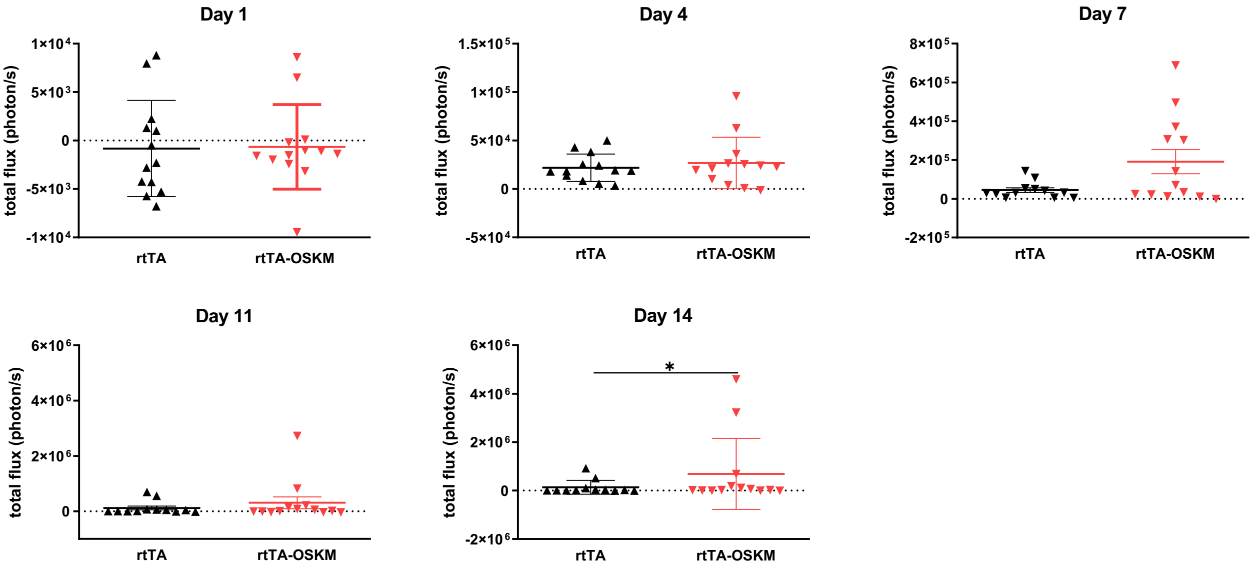

B

Fig. S8 (continued)

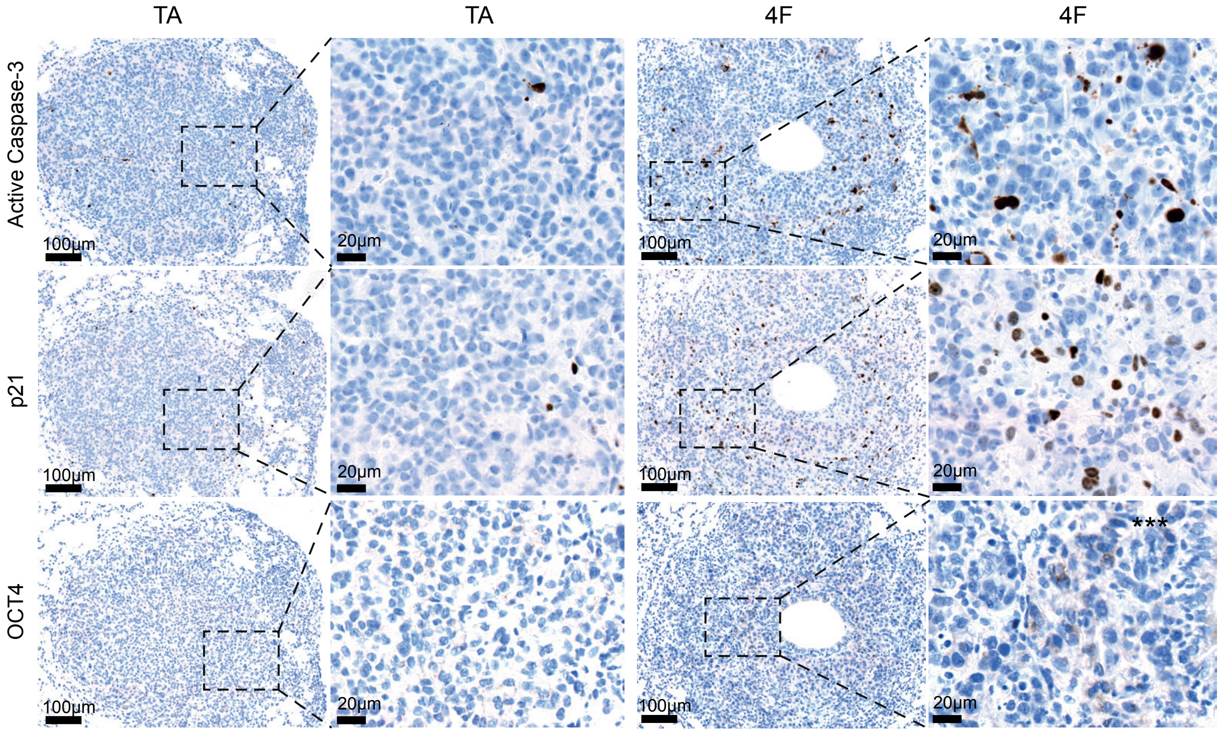

Fig. S8. OSKM expression reduces lung tumor burden in a KrasG12V mouse model. **(A)** Quantification of bioluminescence signal from animals treated with 1 mg/ml doxycycline (i) or non-treated (ii). **(B)** Representative images of immunohistochemistry for active Caspase-3, p21 and Oct4 in TA mice and 4F mice.

Data shown are mean and SD in each individual day. Wilcoxon matched-pairs signed rank test is used to compare the 2 groups on each day. *, $p<0.05$; **, $p<0.01$; ****, $p<0.0001$.

Fig. S9

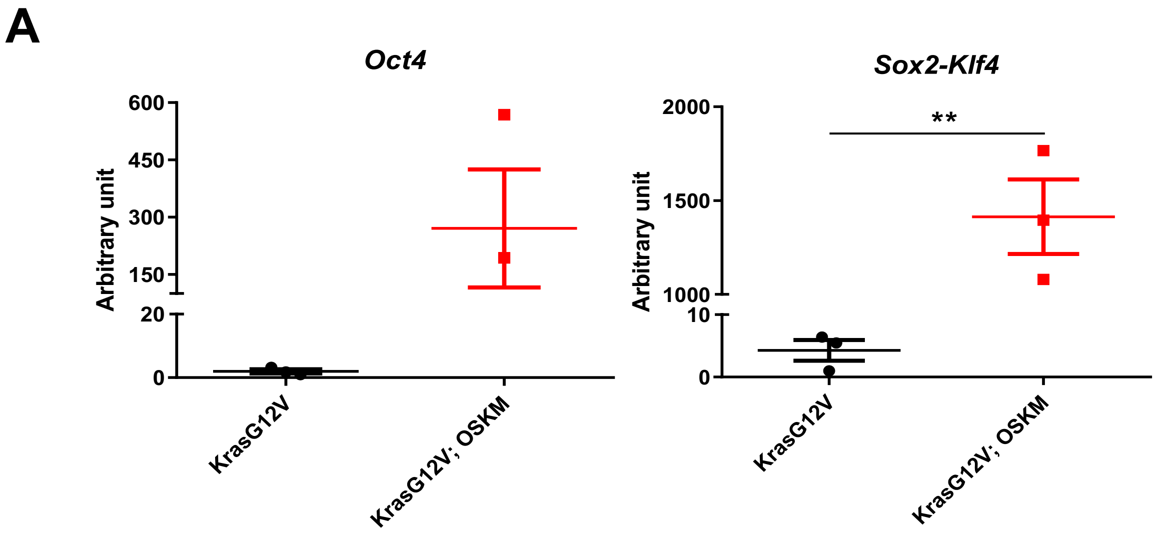
A
Oct4
Arbitrary unit
600
450
300
150
20
0
KrasG12V
KrasG12V; OSKM
Sox2-Klf4
**
2000
1500
1000
10
0

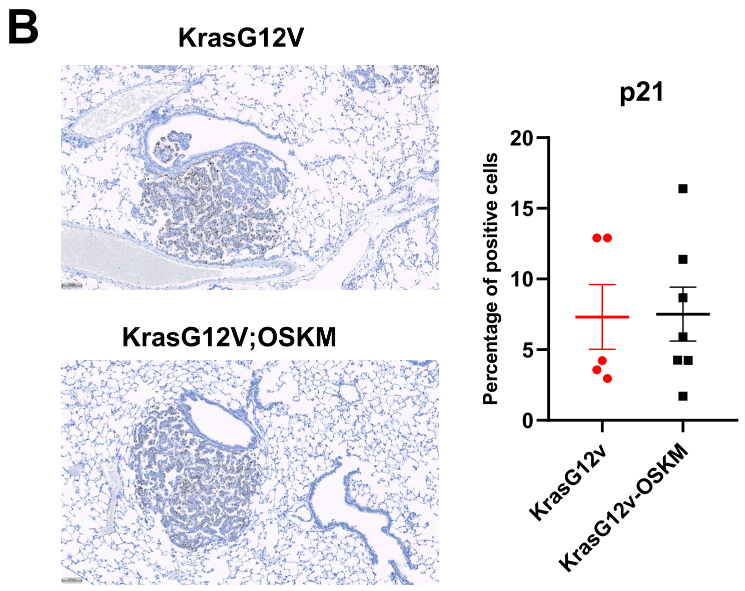
B
KrasG12V
KrasG12V;OSKM
p21
Percentage of positive cells
20
15
10
5
0
KrasG12v
KrasG12v-OSKM

Fig. S9. OSKM expression reduces lung tumor burden in a KrasG12V mouse model. **(A)** Oct4 and Sox2-Klf4 expression by RT-qPCR in lungs of mice that express (KrasG12V;OSKM) or not (KrasG12V) the reprogramming factors. **(B)** Immunohistochemistry for p21 in lungs of KrasG12V and KrasG12V-OSKM mice. Representative images (left) and quantification (right).
